# Energy, nutrient and overall healthiness of processed packaged foods in Fiji, a comparison between 2018 and 2020

**DOI:** 10.1186/s12889-024-18787-1

**Published:** 2024-05-23

**Authors:** Aliyah Palu, Joseph Alvin Santos, Ana Moala Silatolu, Alvina Deo, Colin Bell, Gade Waqa, Jacqui Webster, Briar L. McKenzie

**Affiliations:** 1 The George Institute for Global Health, University of New South Wales, Sydney, NSW 2042 Australia; 2 Pacific Research Centre for the Prevention of Obesity and Non-communicable Diseases, Fiji National University, Tamavua Campus, Suva, Fiji; 3 Ministry of Health, Suva, Fiji; 4 Institute for Health Transformation, Deakin University, Geelong, Australia

**Keywords:** Non-communicable diseases, Nutrition intervention, Fiji, Small island developing states, Salt, Sugar

## Abstract

In Fiji, packaged foods are becoming increasingly available. However, it is unknown if nutrition composition of these foods has changed. This study aims to assess changes in energy, nutrient content and healthiness of packaged foods by comparing data from five major supermarkets in Fiji in 2018 and 2020. Foods were categorised into 14 groups; nutrient composition information was extracted and healthiness assessed using Health Star Rating (HSR). Descriptive statistics and a separate matched products analysis was conducted summarising differences in nutrient content and HSR. There was limited evidence of change in the nutrient content of included products however, there was a small reduction in mean saturated fat in the snack food category (-1.0 g/100 g, 95% CI -1.6 to -0.4 g/100 g). The proportion of products considered healthy based on HSR, increased in the convenience foods category (28.4%, 95% CI 8.3 to 48.5) and decreased in non-alcoholic beverages (-35.2%, 95% CI -43.6 to -26.9). The mean HSR score increased in the fruit and vegetables category (0.1 (95% CI 0.1, 0.2)) and decreased for non-alcoholic beverages (-1.1 (-1.3, -0.9)) and the sauces, dressings, spreads, and dips category (-0.3 (-0.3, -0.2)). Strengthened monitoring of the food supply is needed to improve the healthiness of foods available.

**Supplementary Information:**

The online version contains supplementary material available at 10.1186/s12889-024-18787-1.

## Background

Non-communicable diseases (NCDs) account for 85% of all deaths in Fiji, mostly due to cardiovascular disease and diabetes [1]. Unhealthy diets are a leading risk factor [2], particularly diets characterised by high energy, fat, sodium and sugar intake and low fruit and vegetable intake [3, 4].

Packaged food products are defined as food products that have been sealed within a package before entering the business and remain in that package until sale [5]. Many packaged foods are either processed or ultra-processed, containing additives and/or high in energy, sodium, fat or sugar. Processed foods are defined as foods that have undergone various preservation or cooking methods adding salts, oils and sugars [6]. Ultra-processed foods are defined as “food products typically containing little or no whole foods, are ready to consume or heat up, and are fatty, salty, or sugary and depleted in dietary fibre, protein with various micronutrients and other bioactive compounds” [6]. In Fiji, there has been a rapid increase in the size and number of food retail outlets in the past 20 years, increasing the access to processed packaged foods [7, 8]. Many of these foods are imported [9], with studies reporting increasing imports of foods that are high in sugar such as flavouring extracts of syrups, frozen desserts and bottled and canned soft drinks [10]; and foods that are high in sodium [7] and saturated fats, such as ready meals and packaged chips [3]. Increased accessibility has led to increased consumption of processed packaged foods in Fiji [3] evidenced by the most recent Fiji National Nutrition Survey (2016) reporting high consumption of processed foods such as sausages, canned foods, and cereals [11, 12]. It is thought that the increased accessibility and consumption of these foods in Fiji have contributed to the increasing prevalence of NCDs [13].

Fiji’s Ministry of Health and Medical Services (MoHMS) and the World Health Organization (WHO) released a statement in 2018 calling for healthier diets to address the increasing burden of NCDs [14]. Fiji has committed to meeting the WHO sodium and sugar maximum intake recommendations, with a focus on driving policies to make healthier eating choices the easier choice [15] and influencing consumer knowledge and preference [15]. In 2021, a study found that products in the convenience foods category had the highest level of Na in Fiji, and most packaged foods do not comply with national nutrition labelling regulations [7]. Previous efforts to reduce sodium intake in Fiji have included engaging with the food industry to reduce sodium in processed foods, targeted advocacy efforts, improved health education and hospital programs [16, 17]. In 2014, voluntary sodium targets were proposed for 8 food categories but these were never endorsed by the Fijian Government [7].

Packaged foods have a mandatory nutrition information panel (NIP) on the label which provides product specific nutrient content such as the average quantity of energy in kilojoules or kilocalories, and content of protein, fat, saturated fat, carbohydrates, sugars and sodium (a component of salt) [18]. In Fiji, continued monitoring of the food supply is necessary to better understand the availability of processed foods and their nutrient content [19]. In recent years, Fiji has been exposed to the Health Star Rating (HSR) front of pack labelling scheme used in Australia and New Zealand on some imported, packaged foods. The voluntary HSR system rates the overall healthiness and nutritional content of pre-packaged foods [20]. The healthiness rating is calculated by an algorithm dependent on specific nutrients where products are awarded positive points for certain nutrients (fruit, nut, vegetable, legume, protein and fibre) and negative points for at risk nutrients (total kilojoules, saturated fat, sodium, and total sugars) and the overall food category, products are then given a star rating between 0.5 and 5 [20]. The HSR on packaged foods advises consumers of the overall healthiness of a product so that consumers can make informed decisions. The aim of this study was to determine change over time in the reported energy, nutrient content packaged foods between 2018 and 2020 and the healthiness of packaged food products as determined using the HSR system.

## Methods

### Study design

This study used data that were collected from surveys of processed foods conducted in Fiji’s five largest supermarket chains in 2018 and 2020 using the FoodSwitch program [21]. Each supermarket consented to the collection of publicly available nutrient data shown on the packaging of foods. The FoodSwitch App can be used to monitor the food supply. It captures photos of front and back labels on packaged food products and the nutrient data is then inputted into a database for monitoring and to guide health interventions. The FoodSwitch program has a comprehensive, standardised data collection technology system that collects and collates key data describing packaged foods, and has been used in multiple countries [21].

### Study setting

The five supermarkets were estimated to cover approximately 80% of the processed food supply in Fiji and geographically located in the Central, Western and Northern divisions of Fiji.

### Data extraction

Data were extracted from all packaged foods for human consumption that were available for sale over three months in each supermarket during both data collection periods. During data collection, photos of the front and back labels of a product were taken by trained data collectors. This included the product barcode, nutrition label, ingredient list, product weight and manufacturer details. Images collected were transmitted through a smartphone application to a data management center for data processing. Data was entered into the FoodSwitch database using the packaged food products front-and-back labels by trained data entry personnel. If a product was available in multiple stores, only one of the same products was counted.

### Data processing

Each product was classified according to a standard food categorisation system developed by the Global Food Monitoring Group [22], information on the approach and methods of classification have been published previously [22]. Fourteen major food categories “Bread and Bakery Products”, “Cereal and Grain products”, “Confectionary”, “Convenience foods”, “Dairy”, “Edible Oils and Oil Emulsions”, “Fish and Fish Products”, “Fruit and Vegetables”, “Meat and Meat Products”, “Non-alcoholic Beverages”, “Sauces, Dressings, Spreads and Dips”, “Snack foods”, and “Sugars, Honey, and Related products” and “Special foods”) were included, and then divided into subcategories where applicable (refer to supplementary Table 1). The special foods category includes baby food and protein and diet bars and drinks. However, analysis did not include the *special foods* category due to infrequent contribution of these foods to adult nutrient intake and due to the small number of products in this category.

Food products that did not contribute substantially to energy or nutrient intake (e.g., chewing gum, and herbs and spices, sweeteners), multi-packs, products without a nutrition information panel or where manufacturers are not required to display a nutrient information panel (e.g., baking powders, yeasts and gelatines and cough lollies) were excluded as the nutrition composition of the food product was not available. and products where the nutrition content was reported ‘as prepared’ used nutrient information per 100 g. For example, Milo provides nutrient composition as the powder and as prepared (i.e. with milk) we would use the nutrient information for the powder. Products that were within-year duplicates were also excluded. Packaged foods that were the same product, but different sizes were considered duplicate items in the database, so that each packaged food products were included only once in analysis.

The following nutrients were analysed: energy, total fat, saturated fat, sugars, and sodium. The energy was reported in kJ/100 g, sodium was reported in mg/100 g, and total fat, saturated fats, and sugars were reported in g/100 g. Nutrients reported per serve were converted to per 100 g equivalent where possible and appropriate.

The healthiness of a food product was calculated using the Health Star Rating (HSR) algorithm [23]. The nutrient profiling algorithm rates food and beverages between 0.5 (least healthy) and 5.0 (most healthy) stars [23]. If the HSR was clearly labelled on the packaged food product, the reported HSR was used. If a product did not have a HSR labelled on the pack it was calculated based on the product’s energy, saturated fat, sugars, sodium, protein, and dietary fibre content, following previously established methods [24]. The HSR ranges from 0.5 to 5, with 0.5 being the least healthy and 5 being the most healthy product within the food category according to the system [20]. Products were considered healthy if the HSR was ≥ 3.5 or above.

### Data analysis

The number of products in each major category was recorded in 2018 and again in 2020. For each nutrient/HSR, data were summarised using descriptive statistics.

Mixed effects regression analysis was used to determine changes in mean energy and nutrient content and HSR, as well as the proportion of products with HSR ≥ 3.5 between the two-time points. The analysis was conducted separately by food category, as it was deemed inappropriate to pool categories due to the wide range of nutrients across categories. The analysis of change over time did not include the *special foods* category due to the small number of products in this category.

A separate “matched” analysis based on a product’s name included only products in each food category that were present both in 2018 and 2020 was conducted to assess changes and healthiness within the same packaged food products. This analysis was undertaken to examine if there had been reformulation in packaged products. A matched and unmatched analysis was conducted for each time point and the HSR algorithm was applied with a cut off of 3.5 based on previous studies that have followed a similar approach [23]. Adjustment for multiple comparisons was done using the Benjamini-Hochberg procedure [25]. Statistical analyses were conducted in Stata SE V13.0 for Windows (StataCorp LP, College Station, TX, USA). Alpha was set at a 0.05 significance level.

## Results

**Fig. 1:**
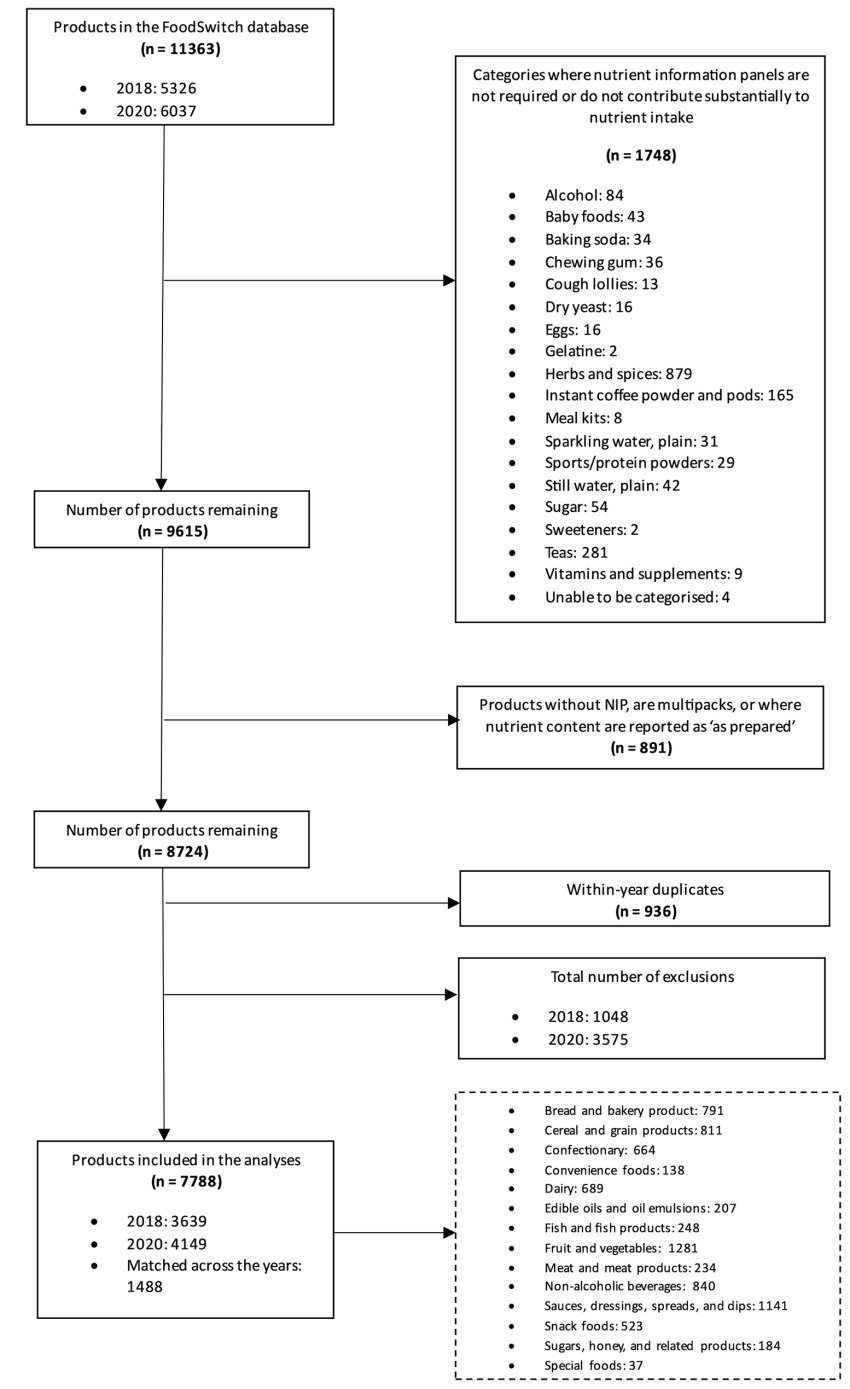
FoodSwitch pre-packaged exclusion and inclusion products

A total of 5326 food products were identified in 2018 and 6037 in 2020. Following the exclusion of products where nutrient information was not available or that did not contribute substantially to nutrient intake, the total number of products included in 2018 was 3,639 and 4,149 in 2020. The number of products within each food category varied, ranging from 138 (convenience foods) to 1,281 (fruits and vegetables) (See Fig. 1).

### Changes in energy and nutrients reported on nutrition labels over time

From 2018 to 2020, there were no changes over time in mean energy content, and total fat, sugar, and sodium content of packaged products across the 13 major food categories examined (see Table 1). There was a small decrease in reported mean saturated fat in the snack food category (-1.0 g/100 g, 95% CI -1.6 to -0.4 g/100 g)). The matched product analysis showed no differences over time in nutrient levels in any food categories (See Table 2).

**Table 1:** Major food categories and nutrient composition

Major food category	Year	N						
			Energy (kJ/100 g)	Total fat (g/100 g)	Saturated fat (g/100 g)	Sugars (g/100 g)	Sodium (mg/100 g)	HSR
**Bread and bakery products**	2018	424	1751.9 (16.6)	14.6 (0.4)	7.1 (0.2)	22.7 (0.7)	417.1 (13.4)	1.8 (0.0)
	2020	367	1735.4 (16.8)	14.5 (0.4)	7.0 (0.2)	22.9 (0.7)	417.1 (13.4)	1.8 (0.0)
	Diff		-16.5 (-36.1, 3.1)	-0.0 (-0.3, 0.2)	-0.0 (-0.2, 0.1)	0.2 (-0.1, 0.4)	-0.0 (-8.7, 8.6)	0.0 (-0.0, 0.0)
**Cereal and grain products**	2018	374	1525.5 (16.6)	8.0 (0.4)	2.3 (0.1)	11.0 (0.5)	255.4 (28.5)	3.5 (0.0)
	2020	437	1527.3 (16.6)	8.0 (0.4)	2.3 (0.1)	10.9 (0.5)	253.3 (28.4)	3.5 (0.0)
	Diff		1.9 (-1.0, 4.7)	-0.0 (-0.1, 0.1)	0.0 (-0.0, 0.0)	-0.1 (-0.2, 0.0)	-2.1 (-7.2, 2.9)	0.0 (-0.0, 0.1)
**Confectionery**	2018	355	1770.7 (23.8)	14.8 (0.6)	9.5 (0.4)	53.9 (0.9)	110.5 (9.2)	1.3 (0.0)
	2020	309	1762.3 (24.5)	14.6 (0.6)	9.4 (0.4)	53.8 (0.9)	110.3 (9.2)	1.2 (0.0)
	Diff		-8.4 (-50.2, 33.4)	-0.2 (-0.4, 0.1)	-0.1 (-0.1, 0.0)	-0.1 (-0.7, 0.6)	-0.2 (-5.9, 5.4)	-0.0 (-0.1. 0.0)
**Convenience foods**	2018	54	670.2 (56.2)	4.6 (0.5)	1.9 (0.3)	3.7 (0.4)	1112.0 (271.6)	2.8 (0.1)
	2020	84	699.3 (54.9)	4.6 (0.5)	1.8 (0.3)	3.7 (0.4)	1153.9 (271.2)	3.0 (0.1)
	Diff		29.1 (-16.3, 74.6)	-0.0 (-0.5, 0.5)	-0.0 (-0.3, 0.2)	-0.0 (-0.3, 0.2)	41.9 (-15.4, 99.3)	0.1 (-0.0, 0.3)
**Dairy**	2018	314	905.9 (24.6)	13.7 (0.5)	9.1 (0.4)	11.7 (0.6)	256.7 (17.2)	2.6 (0.1)
	2020	375	916.7 (24.3)	13.8 (0.5)	9.3 (0.4)	11.6 (0.6)	258.5 (17.1)	2.7 (0.1)
	Diff		10.8 (-7.5, 29.1)	0.1 (-0.0, 0.2)	0.1 (0.0, 0.3)	-0.0 (-0.2, 0.1)	1.9 (-2.0, 5.7)	0.1 (0.0, 0.2)
**Edible oils and oil emulsions**	2018	90	3058.3 (70.8)	84.1 (1.8)	17.8 (1.5)	0.5 (0.2)	171.8 (28.2)	3.2 (0.1)
	2020	117	3205.3 (66.0)	83.9 (1.8)	17.9 (1.5)	0.5 (0.2)	146.0 (26.5)	3.3 (0.1)
	Diff		147.0 (24.1, 269.9)	-0.3 (-0.7, 0.1)	0.1 (-0.3, 0.5)	0.0 (-0.0, 0.0)	-25.8 (-68.7, 17.1)	0.1 (-0.1, 0.3)
**Fish and fish products**	2018	100	576.6 (22.0)	7.1 (0.5)	1.9 (0.1)	1.7 (0.3)	554.1 (62.8)	3.7 (0.1)
	2020	148	570.5 (21.1)	6.3 (0.5)	1.6 (0.1)	1.7 (0.3)	490.7 (59.6)	3.7 (0.1)
	Diff		-6.1 (-36.7, 24.4)	-0.8 (-1.8, 0.2)	-0.3 (-0.5, -0.1)	0.0 (-0.1, 0.1)	-63.4 (-162.6, 35.8)	0.1 (-0.0, 0.1)
**Fruits and vegetables**	2018	577	956.0 (26.8)	10.8 (0.6)	1.8 (0.1)	14.9 (0.7)	366.8 (26.2)	3.7 (0.0)
	2020	704	947.3 (26.6)	10.6 (0.6)	1.8 (0.1)	14.9 (0.7)	363.3 (26.1)	3.8 (0.0)
	Diff		-8.7 (-28.2, 10.8)	-0.2 (-0.4, 0.1)	-0.0 (-0.0, 0.0)	-0.0 (-0.1, 0.1)	-3.4 (-9.9, 3.0)	**0.1 (0.1, 0.2)**
**Meat and meat products**	2018	91	891.5 (28.3)	12.6 (0.6)	7.4 (0.3)	0.8 (0.1)	696.3 (80.3)	2.7 (0.1)
	2020	143	873.3 (27.9)	12.7 (0.6)	7.4 (0.3)	0.8 (0.1)	695.7 (80.3)	2.7 (0.1)
	Diff		-18.2 (-38.5, 2.2)	0.1 (-0.1, 0.3)	0.0 (-0.0, 0.0)	0.0 (-0.0, 0.0)	-0.6 (-1.7, 0.6)	0.1 (0.0, 0.1)
**Non-alcoholic beverages**	2018	407	337.3 (19.7)	0.8 (0.1)	0.8 (0.2)	13.4 (0.8)	51.3 (6.2)	2.9 (0.1)
	2020	433	347.0 (19.6)	0.8 (0.1)	0.7 (0.2)	13.9 (0.8)	52.3 (6.2)	1.8 (0.1)
	Diff		9.7 (-7.3. 26.7)	-0.0 (-0.1, 0.0)	-0.0 (-0.1, 0.0)	0.5 (-0.5, 1.5)	1.0 (-1.2, 3.1)	**-1.1 (-1.3, -0.9)**
**Sauces, dressings, spreads and dips**	2018	520	722.9 (23.4)	9.1 (0.5)	1.9 (0.1)	14.3 (0.6)	1525.8 (90.6)	2.5 (0.0)
	2020	621	742.5 (23.1)	9.1 (0.5)	2.0 (0.1)	14.9 (0.6)	1708.0 (86.3)	2.2 (0.0)
	Diff		19.6 (-2.4, 41.6)	-0.0 (-0.2, 0.2)	0.1 (-0.1, 0.2)	0.6 (-0.2, 1.5)	182.2 (10.0, 354.5)	**-0.3 (-0.3, -0.2)**
**Snack foods**	2018	270	2068.7 (23.5)	25.9 (0.6)	9.6 (0.4)	4.8 (0.3)	647.7 (18.7)	2.2 (0.1)
	2020	253	2040.7 (23.9)	26.6 (0.6)	8.6 (0.4)	4.9 (0.3)	652.9 (18.8)	2.1 (0.1)
	Diff		-28.0 (-88.4, 32.4)	0.7 (-0.2, 1.6)	**-1.0 (-1.6, -0.4)**	0.0 (-0.0, 0.1)	5.2 (-21.7, 32.1)	-0.1 (-0.2, 0.1)
**Sugars, honey and related products**	2018	60	1276.3 (59.2)	4.8 (0.9)	4.1 (0.9)	50.2 (2.8)	56.0 (7.4)	1.7 (0.1)
	2020	124	1299.5 (51.6)	4.5 (0.9)	3.6 (0.8)	53.7 (2.4)	57.6 (7.1)	1.7 (0.1)
	Diff		23.2 (-65.7,112.0)	-0.3 (-0.8, 0.2)	-0.5 (-1.2, 0.2)	3.5 (-1.1, 8.1)	1.6 (-4.6, 7.8)	0.0 (-0.1, 0.2)

*Note Bolded numbers represent significant differences in nutrient composition

### Changes in HSR

There was an increase in mean HSR in packaged fruits and vegetables (0.1 (0.1, 0.2)) and a decrease in mean HSR in non-alcoholic beverages (-1.1 (-1.3, -0.9)) and sauces, dressings, spreads, and dips (-0.3 (-0.3, -0.2)). The matched product analysis showed the same significant changes in HSR in these three product categories (See Table 2).

**Table 2:** Proportion of matched product with a ≥ 3.5 HSR

Matched Product	2018	(%, 95% CI)		2020	(%, 95% CI)		Diff	(%, 95% CI)		pval
Bread and bakery products	5.9	1.8	9.9	5	1.6	8.3	-0.9	-4.7	2.9	0.640
Cereal and grain products	68.7	51.9	85.6	73.6	60.5	86.7	4.9	-6.4	16.1	0.397
Confectionery	3.1	3.1	3.1	3.1	3.1	3.1	0	0	0	0.939
Convenience foods	19.8	4.4	35.2	48.2	29.4	67	28.4	8.3	48.5	0.006
Dairy	18.3	17.4	19.3	18.4	17.3	19.5	0.1	-0.5	0.7	0.786
Edible oils and oil emulsions	49.9	49.6	50.3	48.4	41.5	55.4	-1.5	-8.2	5.2	0.658
Fish and fish products	83.7	73.3	94.2	83.6	74.4	92.7	-0.2	-5.9	5.6	0.951
Fruits, vegetables, nuts and legumes	81.9	81.1	82.6	81.8	81.1	82.6	0	-0.3	0.3	**0.922**
Meat and meat alternatives	17.8	16.7	19	18	17	19	0.2	-0.6	1	0.596
Non-alcoholic beverages	45	36.6	53.4	9.8	5	14.5	-35.2	-43.6	-26.9	**< 0.001**
Sauces, dressings, spreads and dips	51.4	43.7	59.2	34.2	13.9	54.4	-17.2	-35.2	0.7	**0.060**
Snack foods	9.9	2.6	17.2	4.2	0.9	7.4	-5.8	-12.5	1	0.094
Sugars, honey and related products	17.8	17.4	18.3	17.8	17.3	18.3	0	-0.4	0.4	0.922

Statistical analyses were conducted in Stata SE V13.0 for Windows (StataCorp LP, College Station, TX, USA). Alpha was set at a 0.05 significance level

*Note Bolded numbers represent significant differences in HSR

In terms of the proportion of products with HSR ≥ 3.5, the matched analysis showed a similar increase for convenience foods (28.4%, 95% CI 8.3 to 48.5) and a decrease in non-alcoholic beverages (-35.2%, 95% CI -43.6 to -26.9) and the sauces, dressings, spreads, and dips category (-0.3 (-0.3, -0.2)). (See Table 2). There was an increase in unmatched analysis for convenience foods (23.9%, 95% CI 9 to 38.8) and a decrease for non-alcoholic beverages (-37.7%, 95% CI -43.7 to -31,7) (See Table 3).

**Table 3:** Proportion of unmatched product with a ≥ 3.5 HSR

Unmatched	2018	(%, 95% CI)		2020	(%, 95% CI)		Diff	(%, 95% CI)		pval
Bread and bakery products	12.8	9.2	16.5	13.5	10	17	0.7	-2.9	4.3	0.715
Cereal and grain products	54.1	43.6	64.7	62.3	41.8	82.7	8.1	-8	24.3	0.325
Confectionery	3.2	2.1	4.3	3.6	2.4	4.8	0.4	-1	1.8	0.589
Convenience foods	38.8	25.6	51.9	62.7	52.5	72.8	23.9	9	38.8	**0.002**
Dairy	36.2	20.2	52.3	40.4	26.6	54.3	4.2	-9.7	18.1	0.552
Edible oils and oil emulsions	62.8	44.8	80.9	54.2	46	62.4	-8.6	-23.6	6.3	0.259
Fish and fish products	87.6	80.7	94.6	88	82.2	93.7	0.3	-7.1	7.8	0.927
Fruits, vegetables, nuts and legumes	82.2	82.1	82.2	82.2	82.1	82.2	0	0	0	0.998
Meat and meat alternatives	47.7	27.9	67.5	50.3	42.6	58	2.6	-11	16.2	0.708
Non-alcoholic beverages	49.1	43.6	54.7	11.4	8.3	14.6	-37.7	-43.7	-31.7	**< 0.001**
Sauces, dressings, spreads and dips	18.9	17.5	20.3	18.1	17.8	18.3	-0.8	-2.1	0.4	0.183
Snack foods	17.8	17.8	17.8	17.8	17.7	17.9	0	-0.1	0.1	0.740
Sugars, honey and related products	15.3	8	22.6	12	4.3	19.6	-3.4	-11.1	4.4	0.395

Statistical analyses were conducted in Stata SE V13.0 for Windows (StataCorp LP, College Station, TX, USA). Alpha was set at a 0.05 significance level

*Note Bolded numbers represent significant differences in HSR

## Discussion

In Fiji, the total number of packaged food products available to consumers through major supermarkets increased from 2018 to 2020. However, there was no evidence of improvement in nutrient composition of products in most categories between the two time points and the non-alcoholic beverages category became less healthy. Our findings highlight the need for stronger regulation of the food supply in Fiji to aid reformulation of packaged foods, so that healthier options are available for sale, contributing to a healthier food environment.

### Global context

Our findings are in line with global trends in the increasing availability of packaged foods [26–28] and limited nutrient composition change of packaged foods [29]. Similarly to Fiji, upper-middle-income countries (UMIC) and lower-middle-income countries (LMIC) are experiencing increased consumption of packaged foods high in at risk nutrients such as sugar [30] and salt [31, 32]. Our findings contribute to the building narrative about availability and sales of processed packaged foods in the region with studies by Snowdon et al. [3] and Sievert et al. [33] showing increased sales in New Caledonia and Nauru respectively. Similarly, our findings align with studies in the Pacific region that have found increased sales of packaged foods that are often ultra-processed [34] and nutrient poor [35]. On a global scale, our findings are aligned with other FoodSwitch studies conducted in South Africa, that identified the link between packaged processed foods contributing to increased sodium intake with an urgency for stronger regulatory measures [36]. Similar findings in China compared healthiness of food products throughout 2017–2020 and found that like Fiji, China is faced with challenges in regards to reformulation, tax implementation and improved front-of-pack labelling [37]. As such, our findings are in line with other evidence from around the world showing that access to processed foods are increasing with limited evidence of improvements in healthiness.

### Taxes and reformulation – creating healthier options for sale

In this study, the matched food product analysis showed similar results to the unmatched analysis suggesting an absence of reformulation between 2018 and 2020. Our study showed an increase in healthiness for convenience foods (28.4%, 95% CI 8.3 to 48.5), this may be due to small nutrient changes in products through reformulation that contribute to the overall changes in HSR. Conversely, we showed a decrease in the proportion of products with a HSR ≥ 3.5 for non-alcoholic beverages and we found that the average sugar content of these drinks was around 13 g per 100 ml. Studies have shown that 1% of the total mortality rate in Fiji is attributable to SSB consumption, higher than the global average of 0.4% [38]. The Fijian Government introduced a tax on SSB in 2015 of F$0.35/L prod tax & 32% or F$2/L import tariff, whichever is greater [39]. The lack of change in the sugar content of SSB observed in this study suggests further reformulation of SSB is needed. This is concerning given that reformulation, rather than altering purchasing behaviour, is the main mechanism through which SSB taxes have been seen to have positive health impacts in other settings [38]. For example, in the UK the SSB tax is 18p per litre on soft drinks containing between 5 g and 8 g of sugar per 100ml [40]. The UK SSB taxes targeted manufacturers, which has led to subsequent reformulation and extensive health benefits for the public [41] associated with the removal of a total of 45 million kgs of sugar from soft drinks each year [42]. An increase in the SSB tax in Fiji may encourage reformulation of SSB products. Also, given Fiji has a mix of imported [43] and locally manufactured SSBs, applying a tiered tax structure and increasing taxes on locally manufactured SSB, to avoid substitution to locally produced beverages [39], could potentially see greater health impact. In Fiji, there is scope to strengthen existing monitoring and evaluating processes with the aim of reviewing and increasing taxes to decrease the consumption of unhealthy foods and beverages.

### Opportunities for front of pack labelling to aid consumer choice and encourage reformulation

Since 2015, the Consumer Council of Fiji has been lobbying for implementation of front-of-pack labelling so consumers can identify healthier food options [44]. There are a number of front-of-pack labels used globally [45], including endorsement logos seen in products in Northern Europe and Singapore, warning labels such as ‘high in’ labels used on products in Chile [46], South Africa and Mexico and spectrum labels aimed at scoring products by relative healthiness such as Australia and New Zealand’s HSR [44]. The mandatory FoPL in Chile, which aimed to regulate marketing and the sale of products high in sugar, fat and sodium in schools saw a reduction in products with sugar, salt and fats [47]. Reductions were observed in food products labelled as ‘high in sugar’ such as milk based products, beverages, cereals, sweet baked products and sweet and savoury spreads, food products labelled as ‘high in sodium’ such as savoury spreads, cheeses and ready-to-eat meals, and food products labelled as ‘high in saturated fats’ such as savoury spreads post regulations [46, 48]. However, no reductions were observed outside of schools [46] emphasising the need for a FoPL systems, as well as changes that limit availability of unhealthy products in a range of settings rather than only in schools. HSR may be an option in Fiji, given the growing number of products imported from Australia and New Zealand that use the HSR labelling [7]. However, previous evaluations of the HSR in Australia [51] and New Zealand [52, 53] have shown low levels of uptake of the voluntary scheme, and that food industry is more likely to place HSR labels on healthier products. Global evidence suggests that mandatory labels that are easily interpretable (for example warning labels) likely have the biggest influence [54]. A barrier to front-of-pack labelling may be the lack of compliance with Fiji’s national labelling regulations for back-of-pack nutrition labels which state that packaged foods must have nutritional information written in English and clearly state nutritional information per 100 g (100 ml for liquids) for energy, protein, fat and carbohydrates, trans fatty acids, sodium, total sugar, fat, saturated and unsaturated fats [7]. This back-of-pack labelling is used to inform the front-of-pack label. There are potential learnings for Fiji in the implementation of a front-of-pack label from other countries. As with reformulation targets, the voluntary approach for front-of-pack labelling in other countries has been slow, limiting public health impact [55]. For front-of-pack labelling to have an extensive public health impact, there likely needs to be mandatory regulation for and support of food companies to implement front-of-pack labelling across all packaged food products [55].

### Opportunities for stronger reformulation targets

Our findings suggest that due to the limited evidence of change in packaged foods voluntary reformulation targets in Fiji, established in 2014, could be strengthened [7]. These findings align with other voluntary reformulation targets in countries such as the United Kingdom where the salt reduction programme had limited effectiveness [56]. Mandatory reformulation targets for products high in salt, sugar and trans fatty acids may be required [57–59]. It is estimated that health gains from mandatory measures could be 20 times higher than with voluntary interventions [59]. This is also seen in Canada, where foods that are high in sodium, sugar and fats will be required to have a ‘high in’ label on the FoPL by January 2026 [60]. This mandatory regulation is estimated to encourage the food industry to reformulate products [60]. Similarly, in Australia, studies suggest that compared to the voluntary sodium reformulation targets, mandatory sodium reformulation could save more lives annually and achieve greater public health benefits [61]. This reflects, that in Fiji there is a need for mandatory reformulation targets, potentially in a package with taxes, subsidies and front-of-pack labelling, encouraging industry to reformulate food products and making the healthy choice the easier choice for consumers [62].

### Strengths and limitations

A key strength of this study is the approach to data collection in two consecutive time periods using standardised methodology, from major supermarket chains. In addition, Fiji has an evidence based, action oriented Wellness policy that addresses diet among other NCD risk factors, this work highlights areas such as marketing and improving diet that could be focused on further with emphasis on continued monitoring/enforcement [63]. Our study has shown that there is need for continued monitoring and evaluation in Fiji, with the potential to be able to be used for the monitoring and implementation of the Wellness policy and other diet related policies in the future. A limitation was that the data focused specifically on packaged food products and only those food product’s nutrition information was captured. We note that there are potential limitations to the use of the Global Food Monitoring Group food categorisation system and there are other forms of food categorisation systems such as INFORMAS [64], which may have influenced results. Another limitation is the 2 year time period between surveys which was based on other studies conducted in Australia [23], that monitor food supply. In Fiji this timeframe may have been too short, with limited opportunity for changes to be made and then seen at point of sale. However, there are plans to continue monitoring the food supply in Fiji in future years which may yield different results. In addition, there is potential of product duplicates that were included if a product’s barcode had been changed over the two-year period. However, this often suggests that the product was reformulated. A further limitation is the use of the HSR algorithm in this study, which is an Australian/New Zealand system, however, it was deemed appropriate to use this for Fiji, given that Fiji does not currently have a nutrient profiling and front of pack labelling policy in place, and given the imports of packaged foods from Australia and New Zealand that do have this labelling. Furthermore, there are potential limitations of our binary approach to classifying healthiness of products by determining the healthiness of a product with a cut off of 3.5 stars as there are alternative ways to assess ‘healthiness’ such as the use of the NOVA categorisation that focuses on the level of processing of food products [49]. Our study did not use NOVA categorisation as this study is a comparison of two time-points which focuses on specific nutrients outlined by the World Health Organisations ‘Best Buy’ guidelines [50]. A standardised methodology to calculate the HSR was also followed, which has been used and published in other studies and countries [23, 36, 65–67]. Some changes were observed in mean HSR and the proportion of products with HSR ≥ 3.5 over time, despite significant changes in the nutrients focused on in this study. A potential explanation for this is that food manufacturers may have made slight changes in the nutrition composition of food products, including altering fiber and protein content, that would change the HSR rating of a product despite no/limited change in salt, fat, or sugar content.

## Conclusion

The analysis of shop survey data in Fiji identified that there was little change in nutrient content or healthiness based on HSR of packaged food products from 2018 to 2020. An absence of change in nutrient content implies a lack of reformulation, and the need for food policy interventions, such as taxes and subsidies, reformulation targets and FoP labelling, to encourage the formulation of healthier foods available for sale in Fiji. The opportunities identified in this study will be useful in guiding policies for the improvement of the diets and health of Fijians more broadly.

### Electronic supplementary material

Below is the link to the electronic supplementary material.

Supplementary Material 1

Supplementary Material 2

Supplementary Material 3

## Data Availability

The datasets used and/or analysed during the current study available from the corresponding author on reasonable request.

## References

[1] Institute for Health Metrics and Evaluation. Global burden of disease study: University of Washington. 2022 [Available from: https://ghdx.healthdata.org/gbd-2019.

[2] Webster J, Waqa G, Thow AM, Allender S, Lung T, Woodward M, Rogers K, Tukana I, Kama A, Wilson D, Mounsey S, Dodd R, Reeve E, McKenzie BL, Johnson C, Bell C. Scaling-up food policies in the Pacifc Islands: protocol for policy engagement and mixed methods evaluation of intervention implementation. Nutr J. 2022;21(8). 10.1186/s12937-022-00761-5 PMC8807012 35105346

[3] Wendy Snowdon AR, Erica Reeve RLT, Guerrero J, Fesaitu (2013). Processed foods available in the Pacific Islands. Global Health.

[4] GBD Collaborators. Health effects of dietary risks in 195 countries, 1990–2017: a systematic analysis for the global burden of Disease Study 2017. Lancet11. 2019;393. 10.1016/S0140-6736(19)30041-8 PMC6899507 30954305

[5] Victoria Government. Food business classification - Definitions Victoria, Australia: Victoria State Government. 2022 [Available from: https://www.health.vic.gov.au/food-safety/food-business-classification-definitions.

[6] Monteiro CA, Cannon G, Moubarac J-C, Levy RB, Louzada MLC, Jaime PC. The UN decade of nutrition, the NOVA food classification and the trouble with ultra-processing. Public Health Nutr. 2017;5(17). 10.1017/S1368980017000234 PMC10261019 28322183

[7] Shahid M, Waqa G, Pillay A, Kama A, Tukana IN, McKenzie BL, Webster J, Johnson C. Packaged food supply in Fiji: nutrient levels, compliance with sodium targets and adherence to labelling regulations. Public Health Nutr. 2021;24(13). 10.1017/S136898002100224X PMC10195241 34008486

[8] Jimaima T, Schultz PV, Jessie Tuivaga (2004). Fiji National Nutrition Survey.

[9] Andrew NL, Allison EH, Brewer TD, Connell J, Eriksson H, Eurich J, Farmery A, Gephart JA, Golden C, Herrero M, Mapusua K, Seto K, Sharp MK, Thornton P, Thow AM, Jillian Tutuo J. Continuity and change in the contemporary Pacific food systems. Global Food Secur. 2022;32.

[10] Amerita Ravuvu SF, Thow A-M (2017). Wendy Snowdon &amp; Jillian Wate Monitoring the impact of trade agreements on national food environments: trade imports and population nutrition risks in Fiji. Global Health.

[11] Jimaima T, Schultz PV, Jessie Tuivaga (2019). National nutrition survey 2015 report.

[12] Jimaima T, Schultz PV, Ateca Kama, Manager NFNC (2015). Asaeli Naika, Jessie Tuivaga. National nutrition survey 2015.

[13] O’Meara L, Williams SL, Hickes D, Brown P. Predictors of dietary diversity of indigenous food-producing households in rural Fiji. Nutrients. 2019;11(7). 10.3390/nu11071629 PMC6683282 31319537

[14] World Heath Organisation, Ministry of Health, World Health Organization statement on NCD rates in Fiji World Health Organisation. 2018 [Available from: https://www.who.int/westernpacific/about/how-we-work/pacific-support/news/detail/15-06-2018-ministry-of-health-world-health-organization-statement-on-ncd-rates-in-fiji#:~:text=In%202017%2C%20Fiji%20experienced%20almost,31%25%20of%20all%20deaths%20worldwide.

[15] Ministry of Health and Medical Services. National wellness policy for Fiji Fiji: Ministry of Health & Medical Services. 2015 [Available from: https://www.health.gov.fj/wp-content/uploads/2018/03/National-Wellness-Policy-for-Fiji.pdf.

[16] Pillay A, Trieu K, Santos JA, Suku A, Schultz JT, Wate J, Bell C, Moodie M, Snowdon W, Ma G, Rogers K, Webster J. Assessment of a salt reduction intervention on adult population salt intake in Fiji. Nutrients. 2017;9(1350). 10.3390/nu9121350 PMC5748800 29231897

[17] Tora I. Health works on salt reduction Fiji: Fiji News. 2011 [https://fijisun.com.fj/2011/07/25/health-works-on-salt-reduction/.

[18] Food Standards Australia and New Zealand. Nutrition information panels: food standards Australia and New Zealand. 2023.

[19] Tolley HSW, Wate J (2016). Monitoring and accountability for the Pacific response to the non-communicable disease crisis. BMC Public Health.

[20] Australian Government. About Health Star Ratings Canberra: Australian Government. 2023 [http://www.healthstarrating.gov.au/internet/healthstarrating/publishing.nsf/Content/About-health-stars.

[21] The George Institute for Global Heath. FoodSwitch: state of the food supply. The George Institute for Global Health; 2021.

[22] Dunford E, Webster J, Metzler AB, Czernichow S, Ni Mhurchu, Wolmarans P, Snowdon W, L'Abbe M, Li N, Maulik PK, Barquera S, Schoj V, Allemandi L, Samman N, De Menezes EW, Hassell T, Ortiz J, De Ariza JS, Rahman AR, De Núñez L, Garcia MR, Van Rossum C, Westenbrink S, Thiam LM, MacGregor G, Neal B, Food Monitoring Group. International collaborative project to compare and monitor the nutritional composition of processed foods. Eur J Prev Cardiol. 2012;19(6):1326–32. 10.1177/1741826711425777 21971487

[23] Health TGIfG (2020). FoodSwitch: state of the food supply 2020.

[24] Australian Government. The more stars, the healthier Australia. 2017 [Available from: http://www.healthstarrating.gov.au/internet/healthstarrating/publishing.nsf/Content/02728E7D8231ADC5CA257E9600140A63/$File/Factsheet%20for%20Industry.pdf.

[25] Bobbitt Z. A guide to the Benjamini-Hochberg procedure: statology. 2020 [Available from: https://www.statology.org/benjamini-hochberg-procedure/.

[26] Blanco-Metzler A, Vega-Solano J, Franco-Arellano B, Allemandi L, Larroza RB, Saavedra-Garcia L, Weippert M, Sivakumar B, Benavides-Aguilar K, Tiscornia V, Buzarqui GS, Guarnieri L, Meza-Hernández M, Villalba FC, Castronuovo L, Schermel A, L'Abbé MR, Arcand J. Changes in the sodium content of foods sold in four latin American countries: 2015 to 2018. Nutrients. 2021;13(11). 10.3390/nu13114108 PMC8624930 34836362

[27] Lowery CM, Mora-Plazas M, Gomez LF, Popkin B, Taillie LS. Reformulation of packaged foods and beverages in the Colombian Food Supply. Nutrients. 2020;12(11). 10.3390/nu12113260 PMC7692620 33114419

[28] Moz-Christofoletti MA, Wollgast J. Sugars, salt, saturated fat and fibre purchased through packaged food and soft drinks in Europe 2015–2018: are we making progress. Nutrients. 2021;13(7). 10.3390/nu13072416 PMC8308506 34371927

[29] Bernstein JT, Christoforou AK, Weippert M, L’Abbe MR (2020). Reformulation of sugar contents in Canadian prepackaged foods and beverages between 2013 and 2017 and resultant changes in nutritional composition of products with sugar reductions. Public Health Nutr.

[30] Russell C, Baker P, Grimes C, Lindberg R, Lawrence MA. Global trends in added sugars and non-nutritive sweetener use in the packaged food supply: drivers and implications for public health. Public Health Nutr. 2022;26(5). 10.1017/S1368980022001598 PMC10346066 35899782

[31] Baker P, Friel S. Food systems transformations, ultra-processed food markets and the nutrition transition in Asia. Global Health. 2016;12(80). 10.1186/s12992-016-0223-3 PMC5135831 27912772

[32] Cardoso S, Pinho O, Moreira P, Pena MJ, Alves A, Luís Moreira JL, Mendes J, Graça P, Gonçalves C. Salt content in pre-packaged foods available in Portuguese market. Food Control. 2019;106.

[33] Sievert K, Lawrence M, Naika A, Baker P. Processed foods and nutrition transition in the Pacific: regional trends, patterns and food system drivers. Nutrients. 2019;11(6). 10.3390/nu11061328 PMC6628317 31200513

[34] Luiten CM, Steenhuis IH, Eyles H, Ni Mhurchu C, Waterlander WE. Ultra-processed foods have the worst nutrient profile, yet they are the most available packaged products in a sample of New Zealand supermarkets. Public Health Nutr. 2015;19(3). 10.1017/S1368980015002177 PMC10271194 26222226

[35] Brewer TD, Andrew NL, Abbott D, Detenamo R, Faaola EN, Gounder PV, al N, Lui K, Ravuvu A, Sapalojang D, Sharp MK, Sulu RJ, Suvulo S, Tamate JMMM, Thow AM, Wells AT. The role of trade in pacific food security and nutrition. Global Food Security; 2023.

[36] Ndanuko RN, Shahid M, Jones A, Harris T, Maboreke J, Waker A, Raubeheimer D, Simpson SJ, Neal B, Wu JHY , Peters SAE, Woodward M. Projected effects on salt purchases following implementation of a national salt reduction policy in South Africa. Public Health Nutr. 2020;24(14):4614–21. 10.1017/S1368980020005273 PMC10195294 33357250

[37] Li Y, Wang H, Zhang P, Popkin B, D Coyle DH, Ding J, Dong L, Zhang J, Du W, Pettigrew S. Nutritional quality of pre-packaged foods in China under various nutrient profile models. Nutrients. 2022;14(3). 10.3390/nu14132700 PMC9268697 35807879

[38] Mounsey S, Vaqa AK, Cama T, Waqa G, McKenzie BL, Thow AM. Strengthening sugar-sweetened beverage taxation for non-communicable disease prevention: a comparative political economy analysis case study of Fiji and Tonga. Nutrients. 2022;14(1212). 10.3390/nu14061212 PMC8949109 35334867

[39] Teng A, Snowdon W, Tin STW, Genç M, Na’ati E, Puloka V, Signal L, Wilson N. Progress in the Pacific on sugar-sweetened beverage taxes: a systematic review of policy changes from 2000 to 2019. Aust N Z J Public Health. 2021;45(4). 10.1111/1753-6405.13123 34097355

[40] Sasse T, Metcalfe S. Sugar Tax London: Institute For Government. 2022 [https://www.instituteforgovernment.org.uk/article/explainer/sugar-tax#:~:text=The%20levy%20is%20paid%20to,8g%20of%20sugar%20per%20100ml.

[41] Jones A, Wu JHY, Buse K. UK’s Sugar tax hits sweet spot. BMJ Clin Res. 2021;372(463). 10.1136/bmj.n463 33704082

[42] Obesity Evidence Hub. Countries that have taxes on sugar-sweetened beverages (SSBs) Victoria, Australia. 2022 [https://www.obesityevidencehub.org.au/collections/prevention/countries-that-have-implemented-taxes-on-sugar-sweetened-beverages-ssbs.

[43] Lo VYT, Sacks G, Gearon E, Bell C. Did imports of sweetened beverages to Pacific Island countries increase between 2000 and 2015? BMC Nutr. 2021;7(13). 10.1186/s40795-021-00416-4 PMC8135168 34011416

[44] Pettigrew S, Coyle D, McKenzie BL, Vu D, Lim SC, Berasi K, Poowanasatien A, Suya I, Kowal P. A review of front-of-pack nutrition labelling in Southeast Asia: industry interference, lessons learned, and future directions. Lancet Reg Health Southeast Asia. 2022;3(10017). 10.1016/j.lansea.2022.05.006 PMC10305914 37384259

[45] Dunford EK, Ni Mhurchu C, Huang L, Vandevijvere S, Swinburn B, Pravst I (2019). A comparison of the healthiness of packaged foods and beverages from 12 countries using the Health Star Rating nutrient profiling system, 2013–2018. Obes Rev.

[46] Reyes M, Taillie LS, Popkin B, Kanter R, Vandevijvere S, Corvalán C. Changes in the amount of nutrient of packaged foods and beverages after the initial implementation of the Chilean law of food labelling and advertising: a nonexperimental prospective study. PLoS Med. 2020;17(7). 10.1371/journal.pmed.1003220 PMC7386631 32722710

[47] Lindsey Smith Taillie MB, Popkin B, Reyes M, Colchero MA, Corvalán C (2021). Changes in food purchases after the Chilean policies on food labelling, marketing, and sales in schools: a before and after study. Lancet Planet Health.

[48] Taillie LS, Reyes M, Colchero MA, Popkin B, Corvalán C. An evaluation of Chile’s Law of Food labeling and advertising on sugar-sweetened beverage purchases from 2015 to 2017: a before-and-after study. PLoS Med. 2020;17(2). 10.1371/journal.pmed.1003015 PMC7012389 32045424

[49] Monteiro CA, Cannon G, Levy RB, Moubarac JC, Louzada MLC, Rauber F, Khandpur N, Cediel G, Neri D, Martinez-Steele E, Baraldi LG, Jaime PC. Ultra-processed foods: what they are and how to identify them. Public Health Nutr. 2019;22(5). 10.1017/S1368980018003762 PMC10260459 30744710

[50] World Heath Organisation. Tackling NCDs - best buys. Geneva; 2017.

[51] Jones A, Thow AM, Ni Mhurchu C, Sacks G, Neal B (2019). The performance and potential of the Australasian Health Star Rating system: a four-year review using the RE-AIM framework. Aust N Z J Public Health.

[52] Bablani L, Ni Mhurchu C, Neal B, Skeels CL, Staub KE, Blakely TB. Effect of voluntary Health Star Rating labels on healthier food purchasing in New Zealand: longitudinal evidence using representative household purchase data. BMJ Nutr Prev Heath. 2022;5(2). 10.1136/bmjnph-2022-000459 PMC9813620 36619324

[53] Ni Mhurchu C, Eyles H, Choi YY. Effects of a voluntary front-of-pack nutrition labelling system on packaged food reformulation: the Health Star Rating system in New Zealand. Nutrients. 2017;9(8). 10.3390/nu9080918 PMC5579711 28829380

[54] Pettigrew S, Jongenelis M, Maganja D, Hercberg S, Julia C. The ability of nutrition warning labels to improve understanding and choice outcomes among consumers demonstrating preferences for Unhealthy Foods. J Acad Nutr Diet. 2024;124(1):58–e641. 10.1016/j.jand.2023.08.135 37673335

[55] Jones A, Shahid M, Neal B. Uptake of Australia’s Health Star Rating system. Nutrients. 2018;10(8). 10.3390/nu10080997 PMC6115967 30061512

[56] Bandy LK, Hollowell S, Harrington R, Scarborough P, Jebb S, Rayner M. Assessing the healthiness of UK food companies’ product portfolios using food sales and nutrient composition data. PLoS ONE. 2021;16(8). 10.1371/journal.pone.0254833 PMC8336824 34347807

[57] Cobiac LJ, Vos T, Veerman JL. Cost-effectiveness of interventions to reduce dietary salt intake. Heart. 2010;96(23). 10.1136/hrt.2010.199240 21041840

[58] Colman Taylor ACH, Deltetto I, Peacock A, Ha DTP, Sieburg M, Hoang D, Trieu K, Cobb LK (2021). Stephen Jan &amp; Jacqui Webster the cost-effectiveness of government actions to reduce sodium intake through salt substitutes in Vietnam. Arch Public Health.

[59] Goiana-da-Silva F, Cruz-E-Silva D, Allen L, Nunes AM, Calhau C, Rito A, Bento A, Miraldo M, Darzi A. Portugal’s voluntary food reformulation agreement and the WHO reformulation targets. J Glob Health. 2019;9(2). 10.7189/jogh.09.020315 PMC6790236 31656602

[60] Flexner N, Christoforou AK, Bernstein JT, Ng AP, Yang Y, Nilson EAF, Labonté ME, L’Abbe MR. Estimating Canadian sodium intakes and the health impact of meeting national and WHO recommended sodium intake levels: a macrosimulation modelling study. PLoS ONE. 2023;18(5). 10.1371/journal.pone.0284733 PMC10171671 37163471

[61] Trieu K, Coyle DH, Afshin A, Neal B, Marklund M, Wu JHY. The estimated health impact of sodium reduction through food reformulation in Australia: a modeling study. PLoS Med. 2021;18(10). 10.1371/journal.pmed.1003806 PMC8547659 34699528

[62] World Heath Organisation. Tackling NCDs - Best Buys Switzerland. 2017. [https://apps.who.int/iris/bitstream/handle/10665/259232/WHO-NMH-NVI-17.9-eng.pdf.

[63] Ministry of Health and Medical Services. Hopes for higher sugar prices will reduce consumption Fiji: Ministry of Health & Medical Services. 2022 [ https://www.health.gov.fj/hopes-for-higher-sugar-prices-will-reduce-consumption/.

[64] Kumanyika S (2013). INFORMAS (International network for food and obesity/non-communicable diseases research, monitoring and action support): summary and future directions. Obes Rev.

[65] Wong ASC, Coyle D, Wu JHY, Louie JCY. Sodium concentration of pre-packaged foods sold in Hong Kong. Public Health Nutr. 2020;23(15). 10.1017/S1368980020002360 PMC10200669 32744220

[66] Huang L, Ojo AE, Kimiywe JK, Kibet A, Ale BM, Okoro CE, Louie J, Taylor F, Huffman MD, Ojji DB, Wu JHY, Marklund M. Presence of trans-fatty acids containing ingredients in Pre-packaged Foods and the availability of reported trans-fat levels in Kenya and Nigeria. Nutrients. 2023;15(3). 10.3390/nu15030761 PMC9919578 36771466

[67] Lok Yin Chan DHC, Jason HY, Wu, Jimmy Chun Yu Louie (2021). Total and free sugar levels and main types of sugars used in 18,784 local and imported pre-packaged foods and beverages sold in Hong Kong. Nutrients.

