# Supplementary material for: Energy, nutrient and overall healthiness of processed packaged foods in Fiji, a comparison between 2018 and 2020
Source: BMC Public Health. 2024 May 23;24:1383. doi: 10.1186/s12889-024-18787-1 (PMC11112809; doi:10.1186/s12889-024-18787-1)
Supplement: Supplementary file 1 — Supplementary Material 1 [file 12889_2024_18787_MOESM1_ESM.pdf]

Matched and Unmatched Products:

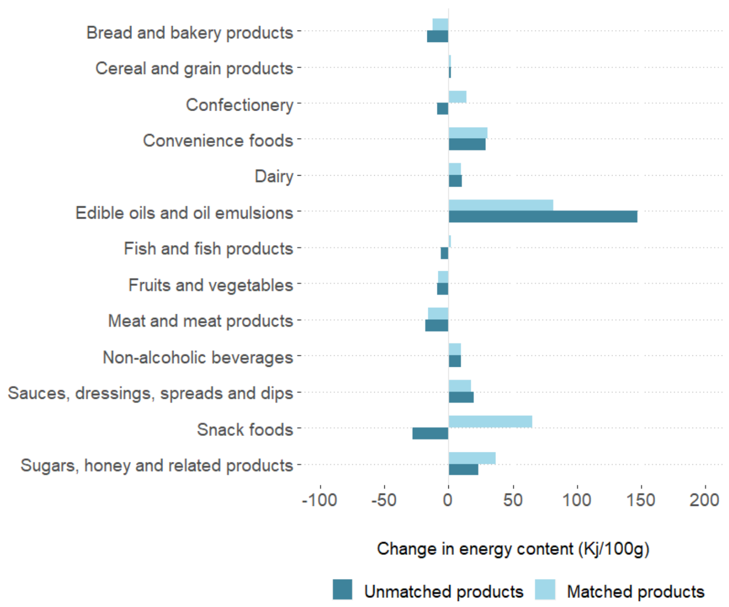

Figure 2. Energy Content (unmatched and matched products)

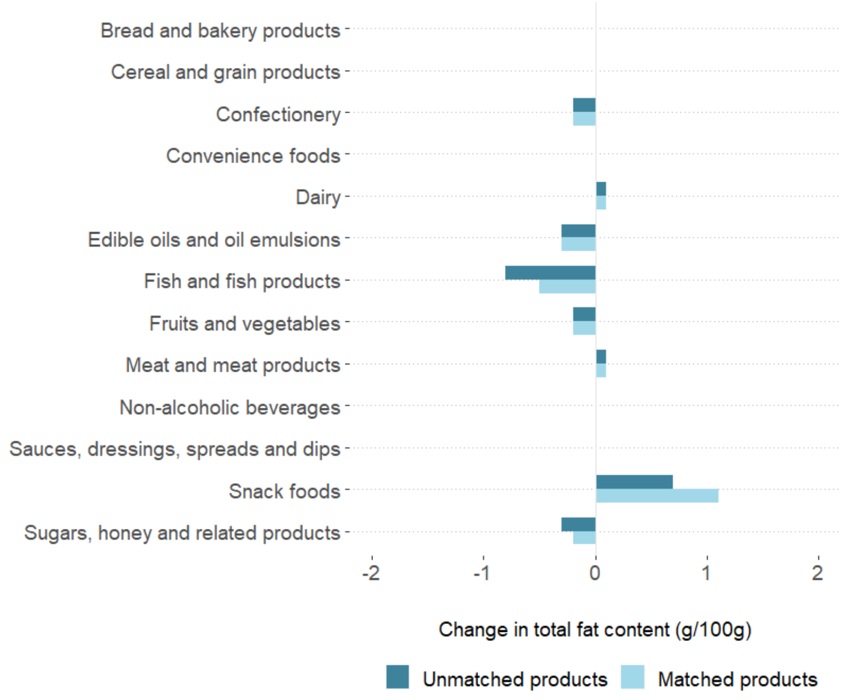

Figure 3. Total Fat content (unmatched and matched products)

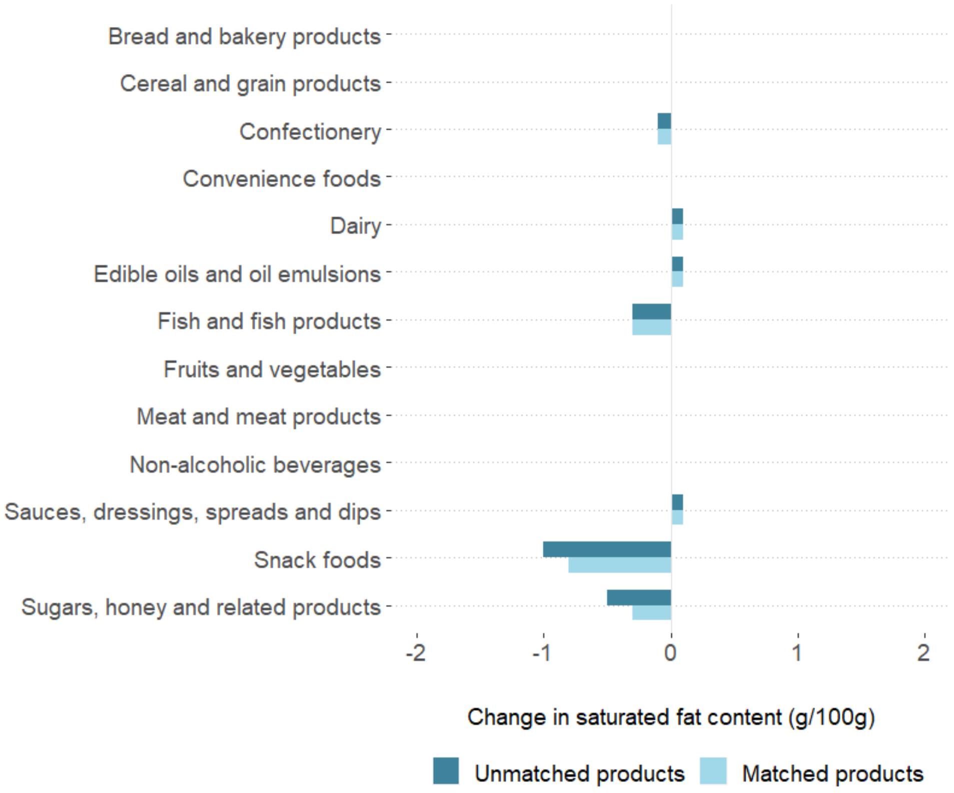

Figure 4. Saturated Fat content (unmatched and matched products)

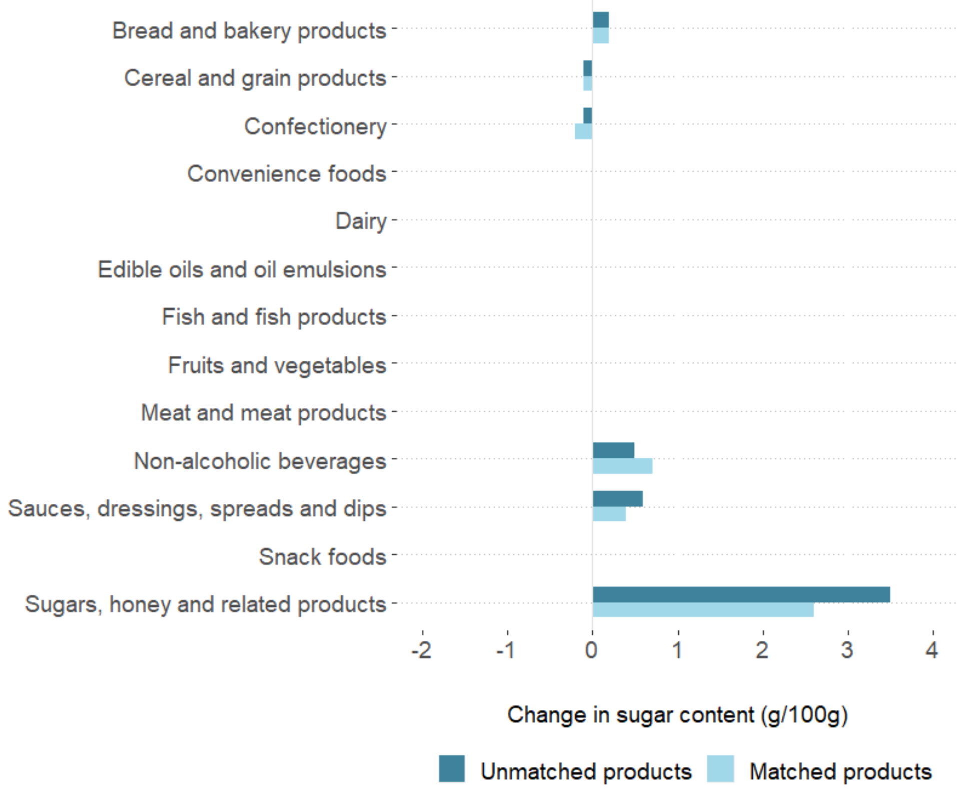

Figure 5. Sugar content (unmatched and match products)

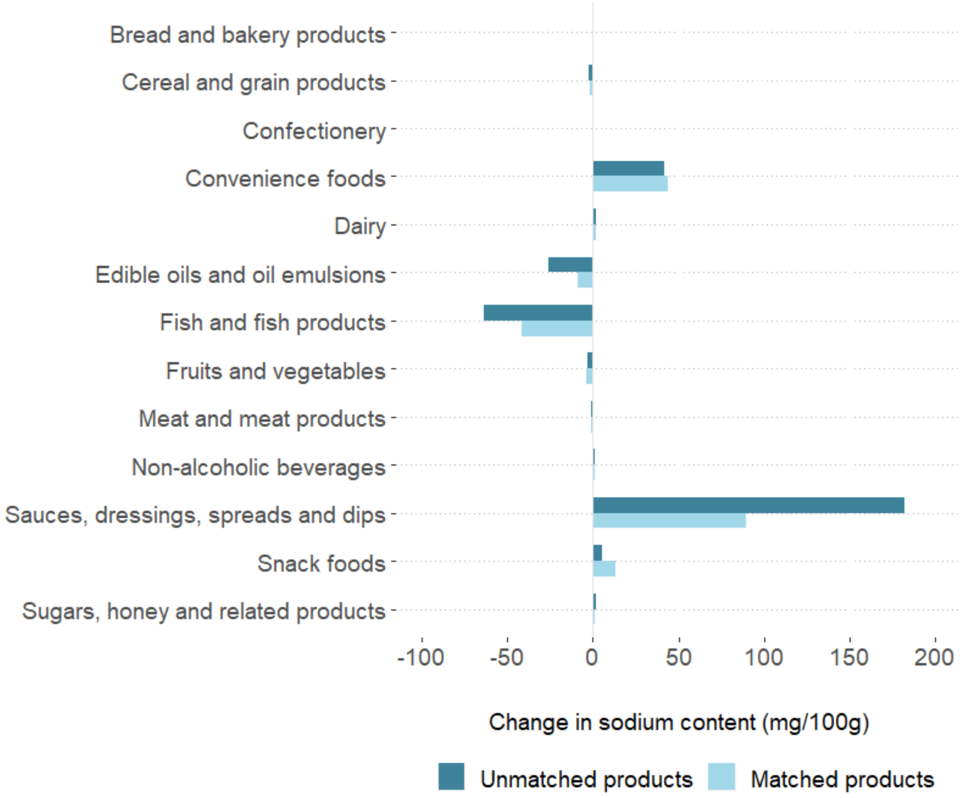

Figure 6. Sodium content (unmatched and matched products)

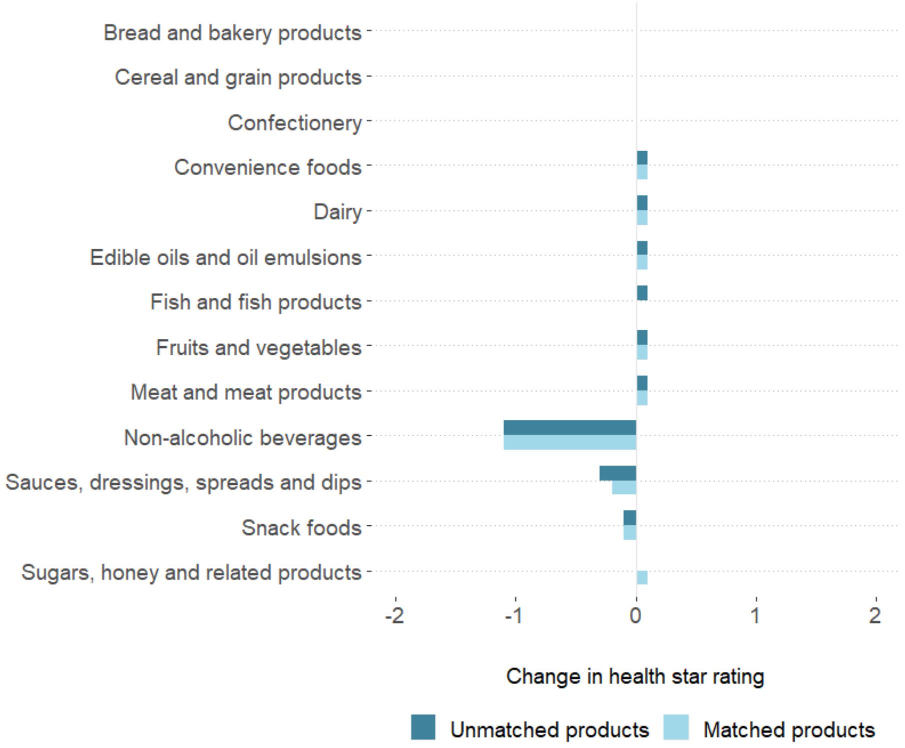

Figure 7. HSR content (unmatched and matched products)
