# Supplementary material for: Energy, nutrient and overall healthiness of processed packaged foods in Fiji, a comparison between 2018 and 2020
Source: BMC Public Health. 2024 May 23;24:1383. doi: 10.1186/s12889-024-18787-1 (PMC11112809; doi:10.1186/s12889-024-18787-1)
Supplement: Supplementary file 2 — Supplementary Material 2 [file 12889_2024_18787_MOESM2_ESM.pdf]

Supplementary Table – Major Food Categories

Major Food Category<br>`	Bread and Bakery Products
	Cereal and Grain Products
	Confectionary
	Convenience Foods
	Dairy Products
	Edible Oils and Oil Emulsions
	Fish and Fish Products
	Fruit and Vegetables
	Meat and Meat Products
	Non-alcoholic Beverages
	Sauces, Dressings, Spreads and Dips
	Snack Foods
	Sugars, Honey, and Related Products
