# Supplementary material for: Energy, nutrient and overall healthiness of processed packaged foods in Fiji, a comparison between 2018 and 2020
Source: BMC Public Health. 2024 May 23;24:1383. doi: 10.1186/s12889-024-18787-1 (PMC11112809; doi:10.1186/s12889-024-18787-1)
Supplement: Supplementary file 3 — Supplementary Material 3 [file 12889_2024_18787_MOESM3_ESM.docx]

STROBE Statement—checklist of items that should be included in reports of observational studies

|  | Item No. | Recommendation | Page  No. | Relevant text from manuscript |
| --- | --- | --- | --- | --- |
| **Title and abstract** | 1 | (*a*) Indicate the study’s design with a commonly used term in the title or the abstract | 2 |  |
|  |  | (*b*) Provide in the abstract an informative and balanced summary of what was done and what was found | 2 |  |
| Introduction | | | |  |
| Background/rationale | 2 | Explain the scientific background and rationale for the investigation being reported | 3 | Non-communicable diseases (NCDs) account for 85% of all deaths in Fiji, mostly due to cardiovascular disease and diabetes (1). Unhealthy diets are a leading risk factor (2), particularly diets characterised by high energy, fat, sodium and sugar intake and low fruit and vegetable intake (3, 4).  Packaged food products are defined as food products that have been sealed within a package before entering the business and remain in that package until sale (5). Many packaged foods are either processed or ultra-processed, containing additives and/or high in energy, sodium, fat or sugar. Processed foods are defined as foods that have undergone various preservation or cooking methods adding salts, oils, and sugars (6). Ultra-processed foods are defined as “food products typically containing little or no whole foods, are ready to consume or heat up, and are fatty, salty, or sugary and depleted in dietary fibre, protein. Various micronutrients and other bioactive compounds” (6). In Fiji, there has been a rapid increase in the size and number of food retail outlets in the past 20 years, increasing the access to processed packaged foods (7, 8). Many of these foods are imported (9), with studies reporting increasing imports of foods that are high in sugar such as flavouring extracts of syrups, frozen desserts and bottled and canned soft drinks (10); and foods that are high in sodium (7) and saturated fats, such as ready meals and packaged chips (3). Increased accessibility has led to increased consumption of processed packaged foods in Fiji (3) evidenced by the most recent Fiji National Nutrition Survey (2016) reporting high consumption of processed foods such as sausages, canned foods, and cereals (11, 12). It is thought that the increased accessibility and consumption of these foods in Fiji have contributed to the increasing prevalence of NCDs (13).  Fiji’s Ministry of Health and Medical Services (MoHMS) and the World Health Organization (WHO) released a statement in 2018 calling for healthier diets to address the increasing burden of NCDs (14). Fiji has committed to meeting the WHO sodium and sugar maximum intake recommendations, with a focus on driving policies to make healthier eating choices the easier choice (15) and influencing consumer knowledge and preference (15). In 2021, a study found that products in the convenience foods category had the highest level of Na in Fiji, and most packaged foods do not comply with national nutrition labelling regulations (7). Previous efforts to reduce sodium intake in Fiji have included engaging with the food industry to reduce sodium in processed foods, targeted advocacy efforts, improved health education and hospital programs (16, 17). In 2014, voluntary sodium targets were proposed for 8 food categories, but these were never endorsed by the Fijian Government (7).  Packaged foods have a mandatory nutrition information panel (NIP) on the label which provides product specific nutrient content such as the average quantity of energy in kilojoules or kilocalories, and content of protein, fat, saturated fat, carbohydrates, sugars, and sodium (a component of salt) (18). In Fiji, continued monitoring of the food supply is necessary to better understand the availability of processed foods and their nutrient content (19). Also, in recent years, Fiji has been exposed to the Health Star Rating (HSR) front of pack labelling scheme used in Australia and New Zealand on some imported, packaged foods. The voluntary HSR system rates the overall healthiness and nutritional content of pre-packaged foods (20). The healthiness rating is calculated by an algorithm dependent on specific nutrients where products are awarded positive points for certain nutrients (fruit, nut, vegetable, legume, protein and fibre) and negative points for at risk nutrients (total kilojoules, saturated fat, sodium, and total sugars) and the overall food category, products are then given a star rating between 0.5-5 (20). The HSR on packaged foods advises consumers of the overall healthiness of a product so that consumers can make informed decisions. |
| Objectives | 3 | State specific objectives, including any prespecified hypotheses | 4 | The aim of this study was to determine change over time in the reported energy, nutrient content of sugar, fats, salt, and energy of packaged foods between 2018 and 2020 and the healthiness of packaged food products as determined using the HSR system. |
| Methods | | | |  |
| Study design | 4 | Present key elements of study design early in the paper | 4 | Study design:  This study used data that were collected from surveys of processed foods conducted in Fiji’s five largest supermarket chains in 2018 and 2020 using the FoodSwitch program (21). Each supermarket consented to the collection of publicly available nutrient data shown on the packaging of foods. The FoodSwitch App can be used to monitor the food supply. It captures photos of front and back labels on packaged food products and the nutrient data is then inputted into a database for monitoring and to guide health interventions. The FoodSwitch program has a comprehensive, standardised data collection technology system that collects and collates key data describing packaged foods, and has been used in multiple countries (21). |
| Setting | 5 | Describe the setting, locations, and relevant dates, including periods of recruitment, exposure, follow-up, and data collection | 5 | Study setting:  The five supermarketsy were estimated to cover approximately 80% of the processed food supply in Fiji and geographically located in the Central, Western and Northern divisions of Fiji. Each supermarket consented to the collection of publicly available nutrient data shown on the packaging of foods. The FoodSwitch App can be used to monitor the food supply. It captures photos of front and back labels on packaged food products and the nutrient data is then inputted into a database for monitoring and to guide health interventions. The FoodSwitch program has a comprehensive, standardised data collection technology system that collects and collates key data describing packaged foods and has been used in multiple countries (21). |
| Participants | 6 | (*a*) *Cohort study*—Give the eligibility criteria, and the sources and methods of selection of participants. Describe methods of follow-up  *Case-control study*—Give the eligibility criteria, and the sources and methods of case ascertainment and control selection. Give the rationale for the choice of cases and controls  *Cross-sectional study*—Give the eligibility criteria, and the sources and methods of selection of participants |  |  |
|  |  | (*b*) *Cohort study*—For matched studies, give matching criteria and number of exposed and unexposed  *Case-control study*—For matched studies, give matching criteria and the number of controls per case | 5 | Data were extracted from all packaged foods for human consumption that were available for sale over three months in each supermarket during both data collection periods. During data collection, photos of the front and back labels of a product were taken by trained data collectors. This included the product barcode, nutrition label, ingredient list, product weight and manufacturer details. Images collected were transmitted through a smartphone application to a data management centre for data processing. Data was entered into the FoodSwitch database using the packaged food products front-and-back labels by trained data entry personnel. If a product was available in multiple stores, only one of the same products was counted. |
| Variables | 7 | Clearly define all outcomes, exposures, predictors, potential confounders, and effect modifiers. Give diagnostic criteria, if applicable |  | Each product was classified according to a standard food categorisation system developed by the Global Food Monitoring Group (22), information on the approach and methods of classification have been published previously (22). Fourteen major food categories “(Bread and Bakery Products”, “Cereal and Grain products”, “Confectionary”, “Convenience foods”, “Dairy”, “Edible Oils and Oil Emulsions”, “Fish and Fish Products”, “Fruit and Vegetables”, “Meat and Meat Products”, “Non-alcoholic Beverages”, “Sauces, Dressings, Spreads and Dips”, “Snack foods”, and “Sugars, Honey, and Related products” and “Special foods”) were included, and then divided into subcategories where applicable (refer to supplementary table 1). The special foods category includes baby food and protein and diet bars and drinks. However, analysis did not include the special foods category due to infrequent contribution of these foods to adult nutrient intake and due to the small number of products in this category minimal contribution to nutrient intake.  Food products that did not contribute substantially to energy or nutrient intake (e.g., chewing gum, and herbs and spices, sweeteners), multi-packs, products without a nutrition information panel or where manufacturers are not required to display a nutrient information panel (e.g., baking powders, yeasts and gelatines and cough lollies), and products where the nutrition content was reported ‘as prepared’ were excluded as the nutrition composition of the food product was not available.. For example, Milo provides nutrient composition as the powder and as prepared (i.e. with milk) we would use the nutrient information for the powder. Products that were within-year duplicates were also excluded. Packaged foods that were the same product, but different sizes were considered duplicate items in the database, however so that each packaged food products were included only once in analysis.  The following nutrients were analysed: energy, total fat, saturated fat, sugars, and sodium. The energy was reported in kJ/100g, sodium was reported in mg/100g, and total fat, saturated fats, and sugars were reported in g/100g. Nutrients reported per serve were converted to per 100g equivalent where possible and appropriate.  The healthiness of a food product was calculated using the Health Star Rating (HSR) algorithm (23). The nutrient profiling algorithm rates food and beverages between 0.5 (least healthy) and 5.0 (most healthy) stars (23). If the HSR was clearly labelled on the packaged food product, the reported HSR was used. If a product did not have a HSR labelled on the pack it was calculated based on the product’s energy, saturated fat, sugars, sodium, protein, and dietary fibre content, following previously established methods (24). The HSR ranges from 0.5 to 5, with 0.5 being the least healthy and 5 being the most healthy product within the food category according to the system (20). Products were considered healthy if the HSR was ≥3.5 or above. |
| Data sources/ measurement | 8* | For each variable of interest, give sources of data and details of methods of assessment (measurement). Describe comparability of assessment methods if there is more than one group |  |  |
| Bias | 9 | Describe any efforts to address potential sources of bias |  |  |
| Study size | 10 | Explain how the study size was arrived at |  |  |

Continued on next page

| Quantitative variables | 11 | Explain how quantitative variables were handled in the analyses. If applicable, describe which groupings were chosen and why |  |  |
| --- | --- | --- | --- | --- |
| Statistical methods | 12 | (*a*) Describe all statistical methods, including those used to control for confounding | 7 | The number of products in each major category was recorded in 2018 and again in 2020. For each nutrient/HSR, data were summarised using descriptive statistics.  Mixed effects regression analysis was used to determine changes in mean energy and nutrient content and HSR, as well as the proportion of products with HSR ≥3.5 between the two-time points. The analysis was conducted separately by food category, as it was deemed inappropriate to pool categories due to the wide range of nutrients across categories. The analysis of change over time did not include the special foods category due to the small number of products in this category.  A separate “matched” analysis based on a product’s name only products in each food category that were present both in 2018 and 2020 was conducted to assess changes and healthiness within the same packaged food products. This analysis was undertaken to examine if there had been reformulation in packaged products. A matched and unmatched analysis was conducted for each time point and the HSR algorithm was applied with a cut of 3.5 based on previous studies that have followed a similar approach (23). any changes in existing products or the addition of new products within categories. Adjustment for multiple comparisons was done using the Benjamini-Hochberg procedure (25). Statistical analyses were conducted in Stata SE V13.0 for Windows (StataCorp LP, College Station, TX, USA). Alpha was set at a 0.05 significance level. |
|  |  | (*b*) Describe any methods used to examine subgroups and interactions |  |  |
|  |  | (*c*) Explain how missing data were addressed |  |  |
|  |  | (*d*) *Cohort study*—If applicable, explain how loss to follow-up was addressed  *Case-control study*—If applicable, explain how matching of cases and controls was addressed  *Cross-sectional study*—If applicable, describe analytical methods taking account of sampling strategy |  |  |
|  |  | (*e*) Describe any sensitivity analyses |  |  |
| Results | | | | |
| Participants | 13* | (a) Report numbers of individuals at each stage of study—eg numbers potentially eligible, examined for eligibility, confirmed eligible, included in the study, completing follow-up, and analysed | 7 | A total of 5326 food products were identified in 2018 and 6037 in 2020. Following the exclusion of products where nutrient information was not available or that did not contribute substantially to nutrient intake, the total number of products included in 2018 was 3,639 and 4,149 in 2020. The number of products within each food category varied, ranging from 138 (convenience foods) to 1,281 (fruits and vegetables) |
|  |  | (b) Give reasons for non-participation at each stage |  |  |
|  |  | (c) Consider use of a flow diagram | 7 |  |
| Descriptive data | 14* | (a) Give characteristics of study participants (eg demographic, clinical, social) and information on exposures and potential confounders |  |  |
|  |  | (b) Indicate number of participants with missing data for each variable of interest |  |  |
|  |  | (c) *Cohort study*—Summarise follow-up time (eg, average and total amount) |  |  |
| Outcome data | 15* | *Cohort study*—Report numbers of outcome events or summary measures over time |  |  |
|  |  | *Case-control study—*Report numbers in each exposure category, or summary measures of exposure |  |  |
|  |  | *Cross-sectional study—*Report numbers of outcome events or summary measures |  |  |
| Main results | 16 | (*a*) Give unadjusted estimates and, if applicable, confounder-adjusted estimates and their precision (eg, 95% confidence interval). Make clear which confounders were adjusted for and why they were included | 8 | From 2018 to 2020, there were no changes over time in mean energy content, and total fat, energy, sugar, and sodium content of packaged products across the 13 major food categories examined (see Table 1). There was a small decrease in reported mean saturated fat in the snack food category (-1.0g/100g, 95% CI -1.6 to -0.4g/100g)). The matched product analysis showed no differences over time in nutrient levels in any food categories |
|  |  | (*b*) Report category boundaries when continuous variables were categorized |  | There was an increase in mean HSR in packaged fruits and vegetables (0.1 (0.1, 0.2)) and a decrease in mean HSR in non-alcoholic beverages (-1.1 (-1.3, -0.9)) and sauces, dressings, spreads, and dips (-0.3 (-0.3, -0.2)). The matched product analysis showed the same significant changes in HSR in these three product categories (See Table 21).  In terms of the proportion of products with HSR ≥3.5, the matched analysis showed a similar increase for convenience foods (28.4%, 95% CI 8.3 to 48.5) and a decrease in non-alcoholic beverages (-35.2%, 95% CI -43.6 to -26.9) and the sauces, dressings, spreads, and dips category (-0.3 (-0.3, -0.2)). (See Table 2). There was an increase in unmatched analysis for convenience foods (23.9%, 95% CI 9 to 38.8) and a decrease for non-alcoholic beverages (-37.7%, 95% CI -43.7 to -31,7) (See Table 3). |
|  |  | (*c*) If relevant, consider translating estimates of relative risk into absolute risk for a meaningful time period |  |  |

Continued on next page

| Other analyses | 17 | Report other analyses done—eg analyses of subgroups and interactions, and sensitivity analyses |  |  |
| --- | --- | --- | --- | --- |
| Discussion | | | | |
| Key results | 18 | Summarise key results with reference to study objectives | 14 | In Fiji, the total number of packaged food products available to consumers through major supermarkets increased from 2018 to 2020. However, there was no evidence of improvement in nutrient composition of products in most categories between the two time points and the non-alcoholic beverages category became less healthy. Our findings highlight the need for stronger regulation of the food supply in Fiji to aid reformulation of packaged foods, so that healthier options are available for sale, contributing to a healthier food environment. |
| Limitations | 19 | Discuss limitations of the study, taking into account sources of potential bias or imprecision. Discuss both direction and magnitude of any potential bias | 18 | A key strength of this study is the approach to data collection in two consecutive time periods using standardised methodology, from major supermarket chains. In addition, Fiji has an evidence based, action-oriented Wellness policy that addresses diet among other NCD risk factors, this work highlights areas such as marketing and improving diet that could be focused on further with emphasis on continued monitoring/enforcement (63). Our study has shown that there is need for continued monitoring and evaluation in Fiji, with the potential to be able to be used for the monitoring and implementation of the Wellness policy and other diet related policies in the future. A limitation was that the data captured foods for sale and not foods consumed. As well as data focused specifically on packaged food products and only those food product’s nutrition information was captured. We note that there are potential limitations to the use of the Global Food Monitoring Group food categorisation system and there are other forms of food categorisation systems such as INFORMAS (64), which may have influenced results. Another limitation is the 2-year time period between surveys which was based on other studies conducted in Australia (23), that monitor food supply. In Fiji this timeframe may have been too short, with limited opportunity for changes to be made and then seen at point of sale. However, there are plans to continue monitoring the food supply in Fiji in future years which may yield different results. In addition, there is potential of product duplicates that were included if a product’s barcode had been changed over the two-year period. However, this often suggests that the product was reformulated. A further limitation is the use of the HSR algorithm in this study, which is an Australian/New Zealand system, however, it was deemed appropriate to use this for Fiji, given that Fiji does not currently have a nutrient profiling and front of pack labelling policy in place, and given the imports of packaged foods from Australia and New Zealand that do have this labelling. Furthermore, there are potential limitations of our binary approach to classifying healthiness of products by determining the healthiness of a product with a cut off 3.5 stars as there are alternative ways to assess ‘healthiness’ such as the use of the NOVA categorisation that focuses on the level of processing of food products (49). Our study did not use NOVA categorisation as this study is a comparison of two time-points which focuses on specific nutrients outlined by the World Health Organisations ‘Best Buy’ guidelines (50). A standardised methodology to calculate the HSR was also followed, which has been used and published in other studies and countries (23, 36, 65-67). Some changes were observed in mean HSR and the proportion of products with HSR ≥3.5 over time, despite significant changes in the nutrients focused on in this study. A potential explanation for this is that food manufacturers may have made slight changes in the nutrition composition of food products, including altering fiber and protein content, that would change the HSR rating of a product despite no/limited change in salt, fat, or sugar content. |
| Interpretation | 20 | Give a cautious overall interpretation of results considering objectives, limitations, multiplicity of analyses, results from similar studies, and other relevant evidence | 14 | Taxes and reformulation – creating healthier options for sale.  In this study, the matched food product analysis showed similar results to the unmatched analysis suggesting an absence of reformulation between 2018 and 2020. Our study showed an increase in healthiness for convenience foods (28.4%, 95% CI 8.3 to 48.5), this may be due to small nutrient changes in products through reformulation that contribute to the overall changes in HSR. Conversely, we showed a decrease in the proportion of products with a HSR ≥3.5 for non-alcoholic beverages and we found that the average sugar content of these drinks was around 13g per 100ml. Studies have shown that 1% of the total mortality rate in Fiji is attributable to SSB consumption, higher than the global average of 0.4% (38). The Fijian Government introduced a tax on SSB in 2015 of F$0.35/L prod tax & 32% or F$2/L import tariff, whichever is greater (39). The lack of change in the sugar content of SSB these drinks between 2018 and 2020 observed in this study suggests further reformulation of SSB is needed. The tax has not encouraged reformulation within a 2-year timeframe and further monitoring is needed. This is concerning given that reformulation, rather than altering purchasing behaviour, is the main mechanism through which SSB taxes haves been seen to have positive health impacts in other settings (38). In comparison, for example, in the UK the SSB tax is 18p per litre on soft drinks containing between 5g and 8g of sugar per 100ml (40). The UK SSB taxes targeted manufacturers, which has led to subsequent reformulation and extensive health benefits for the public (41) associated with the removal of a total of 45 million kgs of sugar from soft drinks each year (42). An increase in the SSB tax in Fiji may encourage reformulation of SSB products. Also, given Fiji has a mix of imported (43) and locally manufactured SSBs, applying a tiered tax structure and increasing taxes on locally manufactured SSB, to avoid substitution to locally produced beverages (39), could potentially see greater health impact. In Fiji, there is scope to strengthen existing monitoring and evaluating processes with the aim of reviewing and increasing taxes to decrease the consumption of unhealthy foods and beverages.  Opportunities for front of pack labelling to aid consumer choice and encourage reformulation.  Since 2015, the Consumer Council of Fiji has been lobbying for implementation of front-of-pack labelling so consumers can identify healthier food options (44). There are a number of front-of-pack labels used globally (45), including endorsement logos seen in products in Northern Europe and Singapore, warning labels such as ‘high in’ labels used on products in Chile (46), South Africa and Mexico and spectrum labels aimed at scoring products by relative healthiness such as Australia and New Zealand’s HSR (44). The mandatory FoPL in Chile, which aimed to regulate marketing and the sale of products high in sugar, fat and sodium in schools saw a reduction in products with sugar, salt and fats (47). Reductions were observed in food products labelled as ‘high in sugar’ such as milk based products, beverages, cereals, sweet baked products and sweet and savoury spreads, food products labelled as ‘high in sodium’ such as savoury spreads, cheeses and ready-to-eat meals, and food products labelled as ‘high in saturated fats’ such as savoury spreads post regulations (46, 48). However, no reductions were observed outside of schools (46) emphasising the need for a FoPL systems, as well as changes that limit availability of unhealthy products in a range of settings rather than only in schools . (49, 50)given the building evidence of the importance of level of processing and health, there is a need to focus on the prevalence of ultra-processed foods available for sale in Fiji going forward. focus on the level of processing for food products in Fiji in the future is crucial to understanding ‘healthiness’ further. Instead, this study used the HSR algorithm for ‘healthiness’ and could be an option HSR seems a viable HSR may be an option in Fiji, given the growing number of products imported from Australia and New Zealand that use the HSR labelling (7). However, Previous evaluations of the HSR in Australia (51) and New Zealand (52, 53) have shown low levels of uptake of the voluntary scheme, and that food industry is more likely to place HSR labels on healthier products. Global evidence suggests that mandatory labels that are easily interpretable (for example warning labels) likely have the biggest influence ((54)). A barrier to front-of-pack labelling may be the lack of compliance with Fiji’s national labelling regulations for back-of-pack nutrition labels which state that packaged foods must have nutritional information written in English and clearly state nutritional information per 100g (100ml for liquids) for energy, protein, fat and carbohydrates, trans fatty acids, sodium, total sugar, fat, saturated and unsaturated fats (7). This back-of-pack labelling is used to inform the front-of-pack label. There are potential learnings for Fiji in the implementation of a front-of-pack label from other countries. As with reformulation targets, the voluntary approach for front-of-pack labelling in other countries has been slow, limiting public health impact (55) If Fiji, is to adopt a FoPL system, mandatory labelling is likely to have the greatest impact. However, from global evidence, other FoPL systems have proven more effective such as warning labels (46). However, a barrier to front-of-pack labelling may be the lack of compliance with Fiji’s national labelling regulations for back-of-pack nutrition labels which state that packaged foods must have nutritional information written in English and clearly state nutritional information per 100g (100ml for liquids) for energy, protein, fat and carbohydrates, trans fatty acids, sodium, total sugar, fat, saturated and unsaturated fats (7). This back-of-pack labelling is used to inform the front-of-pack label. There are potential learnings for Fiji in the implementation of a front-of-pack label from other countries. As with reformulation targets, the voluntary approach for front-of-pack labelling in other countries has been slow, limiting public health impact (55). For front-of-pack labelling to have an extensive public health impact, there likely needs to be mandatory regulation for and support of food companies to implement front-of-pack labelling across all packaged food products (55).  Opportunities for stronger reformulation targets  Our findings suggest that due to the limited evidence of change in packaged foods voluntary reformulation targets in Fiji, established in 2014, could be strengthened (7). These findings align with other voluntary reformulation targets in countries such as the United Kingdom where the salt reduction programme had limited effectiveness (56). Mandatory reformulation targets for products high in salt, sugar and trans fatty acids may be required (57-59). It is estimated that health gains from mandatory measures could be 20 times higher than with voluntary interventions (59). This is also seen in Canada, where foods that are high in sodium, sugar and fats will be required to have a ‘high in’ label on the FoPL by January 2026 (60). This mandatory regulation is estimated to encourage the food industry to reformulate products (60). Similarly, in Australia, studies suggest that compared to the voluntary sodium reformulation targets, mandatory sodium reformulation could save more lives annually and achieve greater public health benefits (61). This reflects, that in Fiji there is a need for mandatory reformulation targets, potentially in a package with taxes, subsidies and front-of-pack labelling, encouraging industry to reformulate food products and making the healthy choice the easier choice for consumers (62). |
| Generalisability | 21 | Discuss the generalisability (external validity) of the study results | 14 | Our findings are in line with global trends in the increasing availability of packaged foods (26, 27) (28) and limited nutrient composition change of packaged foods (29). Similarly to Fiji, upper-middle-income countries (UMIC) and lower-middle-income countries (LMIC) are experiencing increased consumption of packaged foods high in at risk nutrients such as sugar (30) and salt (31, 32). Our findings contribute to the building narrative about availability and sales of processed packaged foods in the region with studies by Snowdon et al (3) and Sievert et al (33) showing increased sales in New Caledonia and Nauru respectively. Similarly, our findings align with studies in the Pacific region that have found increased sales of packaged foods that are often ultra-processed (34) and nutrient poor (35). On a global scale, our findings are aligned with other FoodSwitch studies conducted in South Africa, that identified the link between packaged processed foods contributing to increased sodium intake with an urgency for stronger regulatory measures (36). Similar findings in China compared healthiness of food products throughout 2017-2020 and found that like Fiji, China is faced with challenges in regards to reformulation, tax implementation and improved front-of-pack labelling (37). As such, our findings are in line with other evidence from around the world showing that access to processed foods are increasing with limited evidence of improvements in healthiness. |
| Other information | |  | | |
| Funding | 22 | Give the source of funding and the role of the funders for the present study and, if applicable, for the original study on which the present article is based | 20 | Funding: This research was funded by the National Health and Medical Research Council as part of the Global Alliance for Chronic Diseases program on Scaling up Policy to reduce hypertension and diabetes (APP1169322). BM is supported by a National Heart Foundation of Australia Postdoctoral Fellowship (APP106651). AP is supported by a UNSW Research Training Program (RTP) PhD Scholarship. |

*Give information separately for cases and controls in case-control studies and, if applicable, for exposed and unexposed groups in cohort and cross-sectional studies.

**Note:** An Explanation and Elaboration article discusses each checklist item and gives methodological background and published examples of transparent reporting. The STROBE checklist is best used in conjunction with this article (freely available on the Web sites of PLoS Medicine at http://www.plosmedicine.org/, Annals of Internal Medicine at http://www.annals.org/, and Epidemiology at http://www.epidem.com/). Information on the STROBE Initiative is available at www.strobe-statement.org.
